# Isolation and Characterization of a Single-Stranded DNA Virus Infecting the Marine Diatom *Chaetoceros* sp. Strain SS628-11 Isolated from Western JAPAN

**DOI:** 10.1371/journal.pone.0082013

**Published:** 2013-12-17

**Authors:** Kei Kimura, Yuji Tomaru

**Affiliations:** 1 National Research Institute of Fisheries and Environment of Inland Sea, Fisheries Research Agency, Hatsukaichi, Hiroshima, Japan; 2 Research Fellow of the Japan Society for the Promotion of Science, Tokyo, Japan; Institute of Infectious Disease and Molecular Medicine, South Africa

## Abstract

Diatoms are significant organisms for primary production in the earth's aquatic environment. Hence, their dynamics are an important focus area in current studies. Viruses are a great concern as potential factors of diatom mortality, along with other physical, chemical, and biological factors. We isolated and characterized a new diatom virus (Csp07DNAV) that lyses the marine planktonic diatom *Chaetoceros* sp. strain SS628-11. This paper examines the physiological, morphological, and genomic characteristics of Csp07DNAV. The virus was isolated from a surface water sample that was collected at Hiroshima Bay, Japan. It was icosahedral, had a diameter of 34 nm, and accumulated in the nuclei of host cells. Rod-shaped virus particles also coexisted in the host nuclei. The latent period and burst size were estimated to be <12 h and 29 infectious units per host cell, respectively. Csp07DNAV had a closed circular single-stranded DNA genome (5,552 nucleotides), which included a double-stranded region and 3 open reading frames. The monophyly of Csp07DNAV and other *Bacilladnavirus* group single-stranded DNA viruses was supported by phylogenetic analysis that was based on the amino acid sequence of each virus protein. On the basis of these results, we considered Csp07DNAV to be a new member of the genus *Bacilladnavirus*.

## Introduction

Diatoms (*Bacillariophyta*) account for over 40% of the total marine primary biomass in the oceans and generate most of the organic matter that serves as food for aquatic organisms [1], [2]. Among them, the genus *Chaetoceros* is a major taxonomic group in the case of coastal oceans and approximately 400 species have been described in this genus. The dynamics of diatoms, including *Chaetoceros*, is an important focus area for marine ecology researchers because most diatoms play essential roles as primary photosynthetic producers in various marine environments [3]–[5]. Additionally, several *Chaetoceros* species are harmful to aquaculture, *e.g.*, mortality in caged salmon and damage to the seaweed laver *Porphyra yezoensis*. Recently, several studies reported that the dynamics of diatoms are significantly affected by viruses as well as by diverse physical, chemical, and biological factors [6], [7].

Diatom viruses are classified into 2 groups: single-stranded RNA (ssRNA) viruses and single-stranded DNA (ssDNA) viruses. To date, 5 different species of diatom ssRNA viruses have been reported: *Rhizosolenia setigera* RNA virus (RsetRNAV) [8], *Chaetoceros tenuissimus* RNA virus (CtenRNAV) [9], *C. socialis* f. *radians* RNA virus (CsfrRNAV) [10], *Asterionellopsis glacialis* RNA virus (AglaRNAV) [11], and *C.* sp. strain SS08-C03 RNA virus (Csp02RNAV) [12]. These viruses harbor an ssRNA genome with two ORFs (polyprotein genes) that encode putative replication-related proteins and capsid proteins. Phylogenetic analysis based on the deduced amino acid sequence of the RNA-dependent RNA polymerase domains strongly supported the monophyly of these 3 viruses with a bootstrap value of 100% [10]. Seven ssDNA viruses that infect the centric diatom *Chaetoceros* spp. and the pennate diatom *Thalassionema* have been isolated: *C. salsugineum* DNA virus (CsalDNAV, previously reported as CsNIV) [13], *C. debilis* DNA virus (CdebDNAV) [14], *C. tenuissimus* DNA virus (CtenDNAV) [15], *C. lorenzianus* DNA virus (ClorDNAV) [16], *C.* sp. strain TG07-C28 DNA virus (Csp05DNAV) [17], *C. setoensis* DNA virus (CsetDNAV) (unpublished), and *Thalassionema nitzschioides* DNA virus (TnitDNAV) [11]. Two other diatom viruses, CspNIV and CwNIV, infect *C.* cf. *gracilis* [6] and *C.* cf. *wighamii* [18], respectively, but their nucleic acid types are still unknown. All these diatom viruses have a diameter of 32–38 nm and specifically lyse their respective host diatom species. Although knowledge on diatom viruses has gradually accumulated, more virus isolations and characterizations are required for further understanding diatom host-virus systems that exist in nature. Moreover, the phylogenetic relationships among viruses must be clarified to understand their evolution in the ocean.

In the present study, we have introduced a new ssDNA diatom virus that infects *C.* sp. strain SS628-11, which was isolated from Hiroshima Bay, Japan. In addition, we have performed phylogenetic analysis of ssDNA viruses that infect diatoms.

## Materials and Methods

### Algal cultures and growth conditions

**Figure 1:**
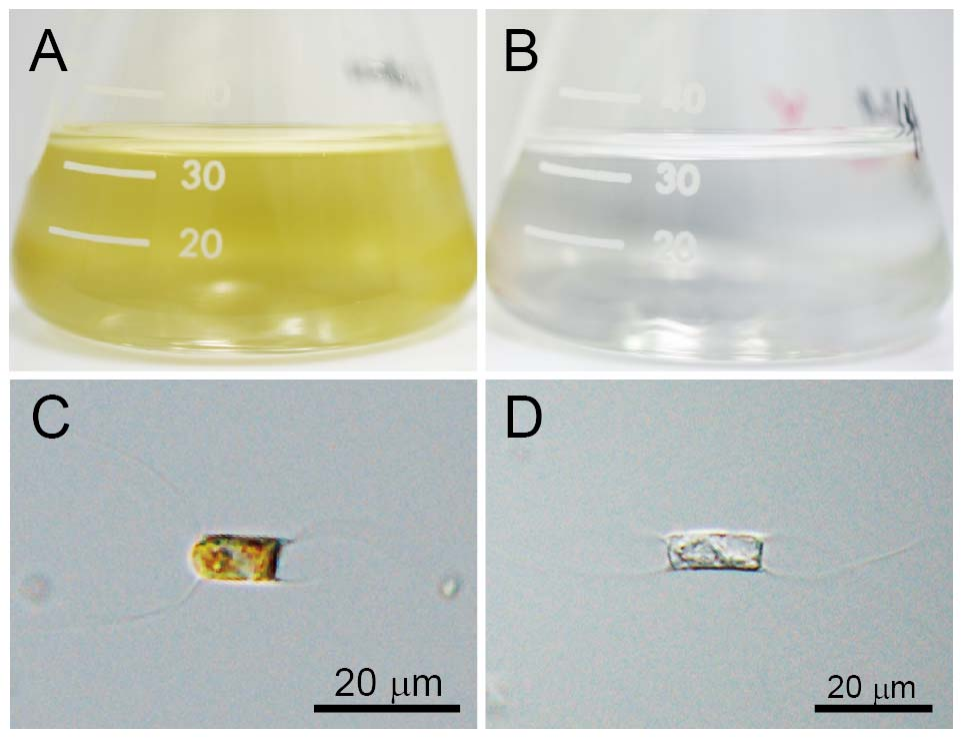
Cultures and micrographs of *Chaetoceros* sp. strain SS628-11 isolated from surface water in Hiroshima Bay, Japan. (A) Cultures without inoculation of Csp07DNAV. (B) Cultures with inoculation of Csp07DNAV at 48 h post-inoculation (hpi). (C) Optical micrograph of an intact cell. (D) Optical micrograph of a Csp07DNAV-inoculated cell at 48 hpi.

The axenic clonal algal strain *C.* sp. SS628-11 (Fig. 1) was isolated from surface water at landing bridge of the National Research Institute of Fisheries and Environment of Inland Sea (FEIS) (34°27.525′N, 132°26.653′E) in Hiroshima Bay, Japan, on June 28, 2011. Algal cultures were grown at 15°C in modified SWM3 medium that was enriched with 2 nM Na_2_SeO_3_ [19] under a 12/12-h light-dark cycle of ca. 110–150 µmol of photons m^−2^•s^−1^ by using cool white fluorescent illumination.

No specific permits were required for the described field studies, as the location is not privately-owned or protected in any way, and the field studies did not involve endangered or protected species.

### Virus isolation

Surface water samples were obtained as described above. The samples were stored at −20°C until analysis. The water samples were filtered through 0.2-µm Dismic-25cs filters (Advantec Toyo, Tokyo, Japan) to remove eukaryotic microorganisms and most bacteria. An aliquot (0.5 mL) of the filtrate was inoculated into an exponentially growing *C.* sp. SS628-11 culture (1 mL), and the cell suspension was then incubated at 20°C by using the lighting conditions described above. Algal cultures that were inoculated with SWM3 served as controls. A *C.* sp. SS628-11 culture that was inoculated with the filtrate exhibited inhibition of algal growth at 2 days post-inoculation (dpi). Cell conditions were observed using an inverted optical microscope (Ti-U; Nikon, Tokyo, Japan). We cloned the pathogen that was responsible for inhibiting the algal growth through 2 extinction-dilution cycles [20], [21] by using lysed cultures of *C.* sp. SS628-11. Briefly, the algal lysate was diluted in modified SWM3 medium over a series of 10-fold dilution steps. Aliquots (100 µL) from each dilution step were added to 8 wells of a 96-well flat-bottom plate (Falcon; Becton, Dickinson and Company, NJ, USA) that contained 150 µL of an exponentially growing host culture. Subsequently, the algal lysate in the well with the highest dilution in the first assay was carried over to the second extinction dilution cycle. The resultant lysate in the final end-point dilution was used as a clonal lysate, for which the probability of the presence of 2 or more viruses (*i.e.*, failure in cloning) was estimated to be <0.0106. Contaminating bacteria were removed from each lysate in the well with the highest dilution in the second assay by filtration through a 0.1-µm polycarbonate membrane filter (Nuclepore), after which the lysate was transferred to an exponentially growing host culture. Bacterial contamination of each lysate was examined using epifluorescence microscopy after staining with SYBR-Gold (Life Technologies, CA, USA). Briefly, the lysate was fixed with 1% glutaraldehyde and a 1.0×10^−4^ dilution of commercial SYBR-Gold stock was added to each fixed sample. The stained samples were filtered through a polycarbonate membrane filter (pore size, 0.2 µm; Nuclepore; Whatman, Kent, UK). Subsequently, the filters were mounted on glass slides with a drop of low-fluorescence immersion oil and then covered with another drop of immersion oil and a cover slip. The slides were viewed at a magnification of 1,000× with an Olympus BX50 epifluorescence microscope (excitation, 470–490 nm; emission, 510–550 nm; dichroic mirror, 505 nm). The resultant axenic lysate was referred to as being a clonal virus (Csp07DNAV) suspension.

### Host range

The interspecies host specificity of the virus Csp07DNAV was tested by the addition of 5% (v/v) aliquots of fresh lysate that had been passed through 0.2-µm filters (Nuclepore) into duplicate cultures of 17 exponentially growing clonal algal strains: *C.* cf. *affinis*, *C. debilis*, *C. lorenzianus*, *C. tenuissimus* 2-6, *C. tenuissimus* 2-10, *C. setoensis*, *C. socialis* f. *radians*, *C.* sp. SS628-11, *C.* sp. TG07-C28, *C.* sp. SS08-C03, *Eucampia zodiacus*, *Asterionellopsis glacialis*, *Thalassiosira rotula* (Bacillariophyceae), *Teleaulax amphioxeia* (Cryptophyceae), *Heterocapsa circularisquama*, *Karenia mikimotoi* (Dinophyceae), and *Heterosigma akashiwo* (Raphidophyceae). Diatoms were cultured at 15°C under the conditions that were described above and the other cultures were maintained at 20°C. Growth and evidence of lysis were monitored in each algal culture by using optical microscopy and were compared with those in control cultures that had been inoculated with SWM3. Cultures that were not lysed at 14 dpi were considered to be unsuitable hosts for the pathogen.

### Virus purification

A 500-mL exponentially growing *C.* sp. SS628-11 culture was inoculated with 5 mL of the virus suspension and lysed. The lysate was passed through 0.4-µm polycarbonate Nuclepore filters (Isopore, Merk, Darmstadt, Germany) to remove cellular debris. Polyethylene glycol 6,000 (Wako Pure Chemical Industries Ltd., Osaka, Japan) was added to the filtrate to achieve a final concentration of 10% (wt/vol), and the suspension was stored at 4°C in the dark overnight. After centrifugation at 57,000× *g* at 4°C for 1.5 h, the pellet was washed with 10 mM phosphate buffer (pH 7.2) and added to an equal volume of chloroform. After vigorous vortexing, the suspension was centrifuged at 2,200× *g* for 20 min at room temperature to remove the chloroform. The water phase was collected and ultracentrifuged at 217,000× *g* for 4 h at 4°C to collect the virus particles. The virus particles were resuspended in 300 µL of ultrapure water, (*i.e.*, virus suspension) and were used for viral genome and protein analyses.

### Transmission electron microscopy

An exponentially growing culture of *C.* sp. SS628-11 was inoculated with Csp07DNAV suspension (5% v/v). As a control, a *C.* sp. SS628-11 culture was inoculated with autoclaved culture medium SWM3. An aliquot of the cell suspension was sampled at 1 dpi. *C.* sp. SS628-11 cells were fixed with 3% glutaraldehyde and 2% paraformaldehyde in SWM3 for 2 h at 4°C. The cells were collected by centrifugation at 2,500× *g* for 5 min at 4°C. After the cell pellets were washed with SWM3, they were embedded into agarose (Type IX-A, Sigma-Aldrich, St Louis, MO) and then post-fixed with 2% OsO_4_ for 2 h on ice. Then, the samples were washed, prestained with 4% uranyl acetate, dehydrated by using an acetone series, and embedded in Spurr's resin (Nisshin EM, Tokyo, Japan). Ultrathin sections were prepared using Ultracut R (Leica, Wetzlar, Germany) and stained with 4% uranyl acetate and 3% lead citrate. The sections were observed under a JEOL JEM-1010 transmission electron microscope (TEM; JEOL, Tokyo, Japan).

Csp07DNAV particles that were negatively stained with uranyl acetate were also observed using TEM. Briefly, the fresh lysate of host culture inoculated with Csp07DNAV was concentrated using an Amicon Ultra-15 30K (Merck) and then mounted on a grid (no. 780111630; Nisshin EM) for 30 s. Excess water was removed using a filter paper (no. 2; Advantec). Subsequently, 4% uranyl acetate was applied for 10 s and any excess dye was removed using a filter paper. After drying the grid, negatively stained Csp07DNAV particles were observed under a TEM at an acceleration voltage of 80 kV. Particle diameters were estimated from the negatively stained images.

### Viral Nucleic acids

RNA and DNA were extracted from the viral pellet by using an RNeasy Plus Mini Kit and a DNeasy Plant Mini Kit (Qiagen, , Valencia, CA), respectively. Each nucleic acid sample was electrophoresed on denatured agarose gels (1.5%; SeaKem® Gold Agarose; TaKaRa Bio, Otsu, Japan) at 50 V for 1 h. The nucleic acids were visualized using SYBR-Gold staining (Life Technologies).

Aliquots (4 µl) of the nucleic acid solution were digested with DNase I (0.5 U•µl^−1^; Takara Bio) at 37°C for 1 h, incubated with RNase A (0.025 µg•µl^−1^; Nippon Gene) at 37°C for 1 h, and digested with S1 nuclease (0.7 U•µl^−1^; Takara Bio) at 23°C for 15 min or boiled at 100°C for 5 min. Nucleic acid extracts that were kept on ice without treatment served as controls. The nucleic acid samples prepared were electrophoresed on 1.5% SeaKem Gold Agarose gel at 50 V for 1 h. The nucleic acids were visualized using SYBR-Gold staining (Life Technologies).

### Viral Genome sequencing

Viral genome DNA sequencing was detected from extracted Csp07DNAV DNA by using GS FLX (Roche, Basel, Schweiz) according to the manufacturer's protocol (Hokkaido System Science, Sapporo, Japan). The sequence data were automatically assembled using GS De Novo Assembler v2.3 (Roche) and manually reassembled with Sequencher v4.9 (Hitachi Soft, Tokyo, Japan). Putative open reading frames were identified using ORF Finder (http://www.ncbi.nlm.nih.gov/gorf/gorf.html). Automated comparisons of the Csp07DNAV sequence with genetic databases were performed using the Basic Local Alignment Research Tool (BLAST) program.

The S1 nuclease-resistant fragment (∼1 kbp) was purified using a QIAquick PCR Purification Kit (Qiagen). It was then blunt ended using T4 DNA Polymerase (TaKaRa Bio) and ligated into the pUC 118 DNA Hin c II/BAP vector (TaKaRa Bio). Subsequently, it was sequenced using an ABI PRISM 3730xl DNA Analyzer (Life Technologies).

### Csp07DNAV proteins

One hundred microliters of virus suspension was mixed with the same volume of denaturing sample buffer (62.5 mM Tris-HCl, 5% 2-mercaptoethanol, 2% sodium dodecyl sulfate [SDS], 20% glycerol, and 0.005% bromophenol blue) and boiled for 5 min. The proteins were then separated using SDS-polyacrylamide gel electrophoresis (PAGE) (12% polyacrylamide gel, 40 mA, 50 min) by employing a mini-Protean system (Bio-Rad, Richmond, CA). The proteins were visualized by using Coomassie brilliant blue stain. Precision Plus Protein Standards (Bio-Rad) were used for size calibration.

### Phylogenetic analysis of viruses

We identified a conserved putative virus protein (VP) 1, VP2 and VP3 encoding regions in the genomic sequence by using BLAST. The deduced amino acid sequence of the corresponding region was compared with those for other viruses. The sequences were automatically aligned using ClustalW [22] and manually refined. Phylogenetic trees were constructed using the maximum likelihood (ML) method with the Jones-Taylor-Thornton matrix that is packaged into MEGA 5 [23]. Amino acid sequences that were used for comparison in the analyses were as follows, with their database accession numbers (refers to the DDBJ database unless otherwise stated): CdebDNAV, AB504376; ClorDNAV, AB553581; CsalDNAV, AB193315; CsetDNAV, AB781089; CtenDNAV, AB597949; Csp05DNAV, AB647334; TnitDNAV, AB781284; and *C.* sp. SS628-11 DNA virus (Csp07DNAV), AB844272.

### Host 18s rDNA sequencing and phylogenetic analysis of host diatoms

DNAs were extracted from *C. lorenzianus* strain IT-Dia51, *Chaetoceros* sp. strain TG07-C28, *C.* sp. strain SS628-11, *C. tenuissimus* strain 2-10, *C. setoensis* strain IT07-C11 and *C. debilis* strain Ch48 by using a DNeasy Plant Mini Kit, and 1 µl each samples were added to 19 µl of PCR reaction mixture that included the 18S ribosomal DNA detection primer set (SR1-SR12) [24]. The PCR reaction was performed as follows: one cycle of 94°C for 2 min, 25 cycles each at 94°C for 30 s, 55°C for 30 s, and 72°C for 1 min, and 72°C for 10 min. Then, nested-PCR was performed under same condition using 1 µl first PCR product as template. Primer sets (SR1b-SR5, SR4-SR9 and SR9-SR12b) were used for nested-PCR [24]. Each amplicon was cloned and sequences were determined using the Sanger dideoxy method on an ABI 3130xl DNA Analyzer. The sequences were automatically aligned using ClustalW and manually refined. Phylogenetic trees were constructed using the maximum likelihood (ML) method with the Tamura-Nei model that is packaged into MEGA 5. The other host diatom DNA sequence of *Thalassionema nizschioides*, X77702, was used for comparison in the analyses.

### Growth experiment

An exponentially growing culture of *C.* sp. SS628-11 (50 mL) was inoculated with Csp07DNAV at a multiplicity of infection of 0.027. A host culture that was inoculated with autoclaved culture medium served as the control. An aliquot of the cell suspension was sampled from each culture at 0, 12, 24, and 36 h post-inoculation (hpi), and the number of host cells and lytic agents were estimated. The reproducibility was checked by performing a similar experiment. Cell counts were carried out with a Fuchs-Rosenthal hemocytometer by using optical microscopy Ti-U without fixation of the samples. The viral titer (*i.e.*, the number of viral infection units) was determined using the extinction dilution method. Briefly, the samples that were used for estimating the viral titer were passed through 0.8-µm polycarbonate membrane filters (Nuclepore) to remove cellular debris. The filtrates were diluted with modified SWM3 medium in a series of 11-fold dilution steps. Aliquots (100 µL) of each dilution were added to 8 wells of a 96-well flat-bottom plate that contained 150 µL of an exponentially growing culture of host algae. The cell culture plates were incubated at 15°C under a 12-h L∶12-h D cycle of 130–150 µmol photons m^−2^•s^−1^ with cool white fluorescent illumination and were monitored by optical microscopy over 14 d for the occurrence of culture lysis. Culture lysis due to virus infection was usually observed as near-complete destruction of the host cells in a well. By using a BASIC program, we calculated the viral titer from the number of wells in which algal lysis occurred [25].

## Results and Discussion

### Isolation of the viral pathogen and determination of its host range

The isolated virus retained its lytic activity (Fig. 1A, B) and was serially transferable to exponentially growing *C.* sp. SS628-11 cultures. The cytoplasmic and photosynthetic pigments in virus-infected SS628-11 cells were found to be degraded compared to those in healthy cells (Fig. 1C and D). The host range of the virus was tested using 17 phytoplankton strains, including 13 diatom strains. Csp07DNAV caused lysis in only its single host strain and not in any other microalgal species that was tested, which indicated that the virus had high infection specificity (Table 1).

**Table 1:** Infection Specificities of Csp07DNAV against 17 strains of marine phytoplankton.

Family	Species	Strain code	Temperature	Strains lysed by CtenDNAV06
Bacillariophyceae	*Chaetoceros sp.*	SS628-11	20	++
	*Chaetoceros sp.*	TG07-C28	15	−
	*Chaetoceros sp.*	SS08-C03	15	−
	*Chaetoceros* cf. *affinis*	IT07-C40	15	−
	*C. debilis*	Ch48	15	−
	*C. lorenzianus*	IT-DiaD51	15	−
	*C. tenuissimus*	2-6	15	−
	*C. tenuissimus*	2-10	15	−
	*C. setoensis*	IT07-C11	15	−
	*C. socialis* cf. *radians*	L-4	15	−
	*Eucampia zodiacus*	EzB	15	−
	*Asterionellopsis glacialis*	Ast K25	15	−
	*Thalassiosira rotula*	It-Dia1	15	−
Cryptophyceae	*Teleaulax amphioxeia*	Tel5W4	20	−
Dinophyceae	*Heterocapsa circularisquama*	HU9433-P	20	−
	*Karenia mikimotoi*	KmY7	20	−
Raphidophyceae	*Heterosigma akashiwo*	HaSS12-1	20	−

+: lysed, −: not lysed.

### Morphological features

**Figure 2:**
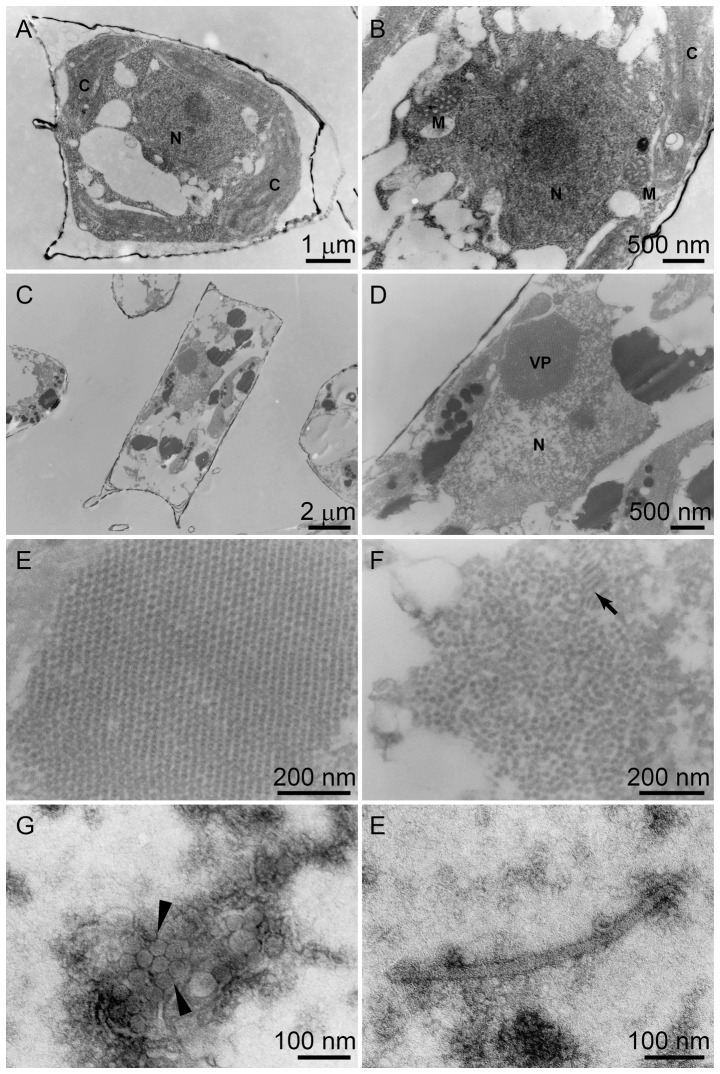
Transmission electron micrographs of ultrathin sections of *Chaetoceros* sp. strain SS628-11 and negatively stained Csp07DNAV particles. (A, B) A healthy cell. (A) A whole cell. (B) Higher magnification of a nucleus. (C–F) Cells that were infected with Csp07DNAV at 2 dpi. (C) A whole cell. (D) Higher magnification of a nucleus with virus-like particles (VLPs). The arrow denotes the accumulation of VLPs. (E) Crystalline array aggregation of VLPs in an infected host nucleus. (F) Random aggregation of VLPs with rod-shaped particles in an infected host nucleus. Arrows: rod-shaped particles. (G) Negatively stained Csp07DNAV particles in culture lysate. Arrow heads: virus particles. (H) Negatively stained rod-shaped particles in culture lysate. N, nucleus; C, chloroplast; M, mitochondrion; and VP, virus particle.

Thin sections of healthy *C.* sp. SS628-11 cells exhibited cytoplasmic organization and frustules that are typical of diatom cells (Fig. 2A). Electron micrographs of thin-sectioned *C.* sp. SS628-11 cells with the virus at 24 hpi exhibited the presence of virus-like particles (VLPs) that were assembled in the host nucleus (Fig. 2C and D). Paracrystalline arrays and random aggregation of VLPs were also observed in the host nucleus (Fig. 2E and F). Paracrystalline arrays of VLPs have been previously reported for the pennate diatom virus TnitDNAV (Tomaru et al. 2012). In contrast, no VLPs were detected in healthy control cells, which included the nucleus (Fig. 2A and B).

Further, VLPs were observed in culture lysates by using negative staining. They were icosahedral, with a diameter of 34±2 nm (observed particle number: *n* = 77), and lacked a tail and an outer membrane (Fig. 2G). Because the algacidal pathogen was transferable to a fresh algal culture and the VLPs were observed in the lysed culture and not in healthy cultures, we concluded that the VLPs that we observed in the infected cells and in the algal lysates belonged to a previously undescribed virus that is pathogenic to *C.* sp. strain SS628-11.

In addition to the VLPs, rod-shaped structures that were 27 nm wide were observed in the host nuclei (Fig. 2F). Similar structures in the virus-infected host nuclei have been reported for other *Chaetoceros* viruses: ClorDNAV, CtenDNAV, and Csp05DNAV [15]–[17]. In these previous studies, rod-shaped structures were not observed in the viral lysate. Therefore, these particles in the host nucleus were considered to be the precursors of mature virions [16], [26]. However, in the present study, many rod-shaped structures (26±1 nm wide and 486±88 nm long [observed particle number: *n* = 11]) were noted in the negatively stained viral suspensions (Fig. 2H). The width of these structures was coincident with rod-shaped materials on thin sections. Since rod-shaped structures were observed in the negative stained lysate, they were considered to have been released from virus-infected cells with mature particle-shaped virions. We hypothesize that the rod-shaped material was a coinfecting virus. Further analyses are required to clarify the role of these materials and the relationships among icosahedral virions.

### Viral genome and proteins

**Figure 3:**
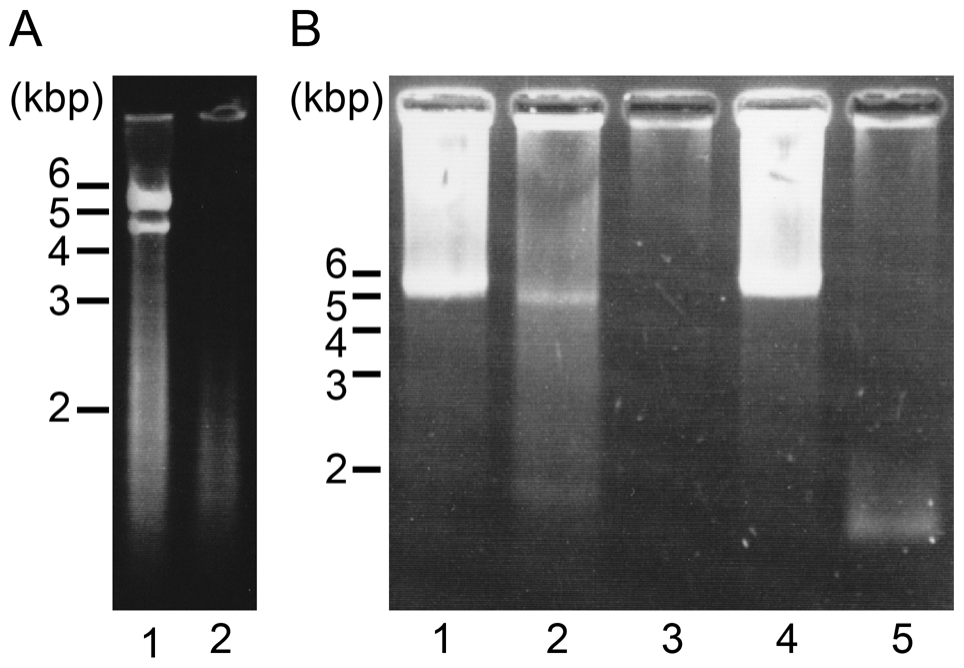
Nucleic acids analysis of Csp07DNAVgenome. (A) Csp07DNAV genome. Extracts of DNA (lane 1) and RNA (lane 2). (B) Nucleic acids of Csp07DNAV without treatment (lane 1), 100°C for 5 min (lane 2), treated with DNase I (lane 3), RNase A (lane 4), and S1 nuclease (lane 5). The samples were electrophoresed on a formaldehyde-agarose gel.

On performing denaturing agarose gel electrophoresis, since nucleic acid bands were observed for the DNA extraction sample but not the RNA extraction sample, the virus genome was considered to be composed of DNA (Fig. 3A). The major nucleic acid bands were at ca. 4.8 and 5.5 kb. After heat treatment at 100°C for 5 min, the intensity of the larger band decreased while that of the smaller band increased (Fig. 3B; lane 2). All the bands were sensitive to DNase I but not to RNase A (Fig. 3B; lanes 3 and 4, respectively). Therefore, these results supported our belief that the viral genome comprises DNA. In addition, the genome was digested with S1 nuclease. However, dsDNA of about 1 kbp remained undigested (Fig. 3B; lane 5). This result is typical of a covalently closed circular ssDNA genome that contains a partially dsDNA region, as observed for all diatom DNA viruses that have been reported, including CsalDNAV, CdebDNAV, ClorDNAV, CtenDANV, and Csp05DNAV [13]–[17].

**Figure 4:**
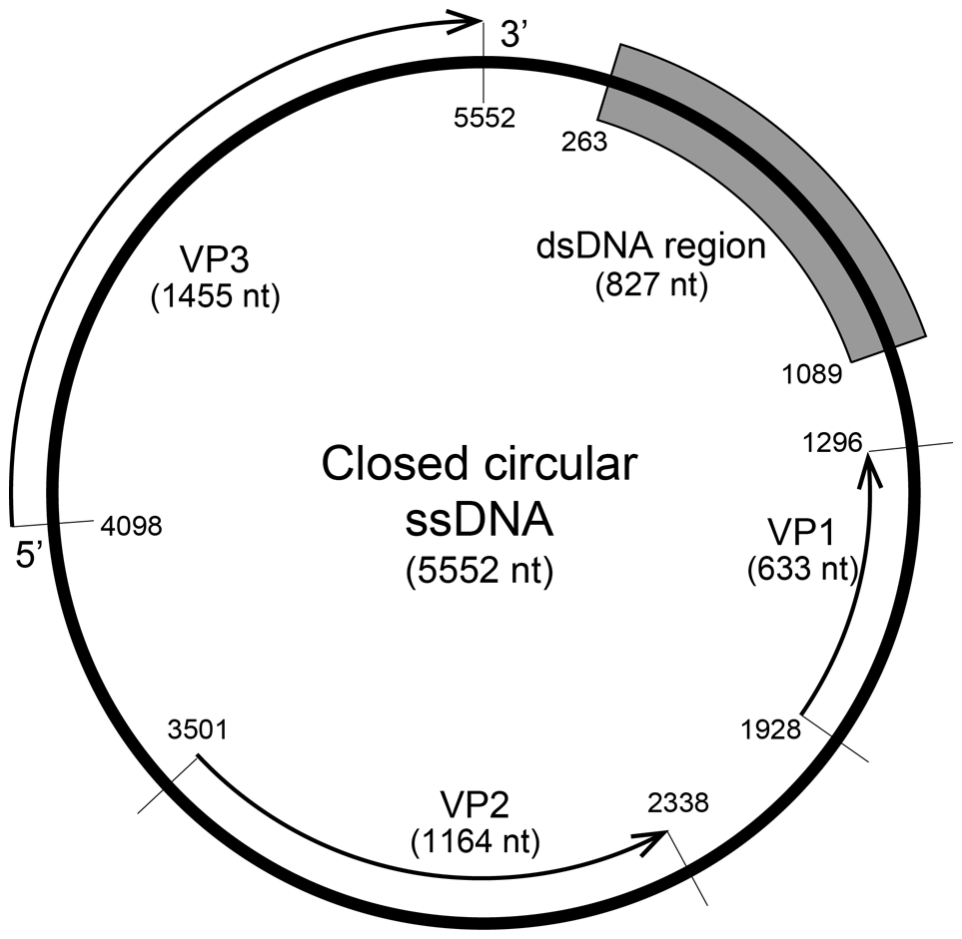
Schematic genomic structure of Csp07DNAV. The arrows indicate the locations for ORFs VP1 to VP3, and the shaded box is the partially double-stranded DNA region.

The following experiments were conducted to confirm the genomic structure of Csp07DNAV (see Materials and methods). Full sequencing of the CtenDNAV genome showed that the ssDNA and dsDNA regions were 5,552 and 827 nt in length, respectively (DDBJ accession no. AB844272). The sequences indicated that the genome included covalently closed circular DNA. Based on these data, we concluded that the viral genome consists of a single-strand of covalently closed circular DNA (5,552 nt) that is partially double stranded (827 bp) (Fig. 4).

The genome includes at least 2 major ORFs (≥300 amino acids [AAs]) and 1 short ORF, which are designated VP1–VP3 (Fig. 4). The largest ORF, VP3, with 484 AAs was predicted to encode a replication protein and showed high similarity with the putative protein of Csp05DNAV (E-value: 1e-178) and other centric diatom DNA viruses. It showed low similarity with the replication protein of a rodent stool-associated circular genome virus (E-value: 2e–4) and the replication protein of bat circovirus (E-value: 3e–4), which are both ssDNA viruses (family Circoviridae, genus Circovirus; Todd et al. 2,000). Other ORFs, VP1 (210 AAs) and VP2 (387 AAs), also had similarities with the putative proteins of ClorDNAV (E-value: 4e–17) and CsalDNAV (E-value: 3e–48), respectively. VP2 (387 AA) was predicted to be a structural protein of the virus based on our preliminary experiment (our unpublished data).

**Figure 5:**
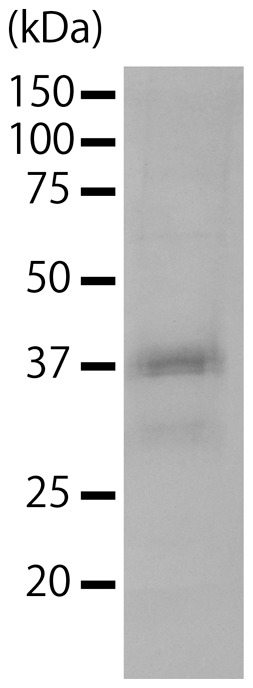
Structural protein of Csp07DNAV.

The sizes and numbers of structural proteins of the virus particles were determined using SDS-PAGE. Csp07DNAV expressed at least 1 protein at 37.0 kDa (Fig. 5). The numbers and sizes of the major proteins of Csp07DNAV were similar to those of the ssDNA diatom virus CtenDNAV, which has a 38.5 kDa protein [15]. In contrast, the molecular weight of VP2 protein that was predicted from the virus genome was ca. 42 kDa. The molecular weights of structural proteins, as determined by SDS-PAGE, might change because of the difference in their electric properties due to protein modification.

Based on the present results, genome type, genomic structure, sequences, and protein size, Csp07DNAV was considered to be similar to the genus *Bacilladnavirus*, which is a genus that has been newly accepted by the International Committee on the Taxonomy of Viruses (ICTV) (Table 2).

**Table 2:** Basic characteristic of single-stranded DNA diatom viruses that have been isolated.

Virus	Host	Isolation field	Isolation year	Particle size (nm)	Particle assembly site and aggregation pattern	Rod shaped particles	Major proteins (kDa)	Latent period	Burst size (infectious units cell-1)	Genomic structure	Genome length (nt)	Complementary fragments (nt)	Data base	Reference
CdebDNAV	*Chaetoceros debilis*	Ariake Sound	2003–2005	32	nucleus, random	nd	37.5, 41	12–24	55	nd	ca. 7 kb not fully sequenced	nd	AB504376	Tomaru et al. 2008
ClorDNAV	*C. lorenzianus*	Hiroshima Bay	2007	34	nucleus, random, ring formation	Yes	<225*	48	2.2×104	covalently closed circular	5,813	979	AB553581	Tomaru et al. 2011c
CsalDNAV	*C. salsugineum*	Ariake Sound	2003	38	nucleus, random	nd	43.5, 46	12–24	325	covalently closed circular	6,000	997	AB193315	Nagasaki et al. 2005
CsetDNAV	*C. setoensis*	Hiroshima Bay	2007	33	nucleus, random	Yes	31, 37	48	2.0×104	covalently closed circular	5,836	67, 70, 72, 76, 90, 107, 109, 145		Tomaru et al.
CtenDNAV	*C. tenuissimus*	Ariake Sound	2005	37	nucleus, random	Yes	38.5	96	320	covalently closed circular	5639	875	AB597949	Tomaru et al. 2011a
Csp05DNAV	C. sp. strain TG07-C28	Ago Bay	2008	33	nucleus, random	Yes	40, 75	<24	nd	covalently closed circular	5785*	890*	AB647334	Toyoda et al. 2012
Csp07DNAV	C. sp. strain SS628-11	Hiroshima Bay	2011	33.7	nucleus, random, crystalline array	Yes	38.5	<12	29.1	covalently closed circular	5552	827		This study
TnitDNAV	*Thalassiosira nitzschioides*	Ariake Sound	2010	35	nucleus, paracrystalline array	nd	nd	nd	nd	covalently closed circular	5573*	ca. 600, not sequenced		Tomaru et al. 2012

* unpublished data.

The knowledge available on the relationships between diatoms and their infectious viruses is not extensive. In the present study, we isolated Csp07DNAV from Hiroshima Bay. ClorDNAV and CsetDNAV were also isolated from the same location. Recent studies on diatom and virus dynamics have suggested that viruses play important roles in regulating diatom population dynamics [7]. Additionally, it is well known that diatom blooms often include multiple species of *Chaetoceros* [5]. Therefore, various diatom host-virus systems may exist in the coastal waters surrounding Japan.

### Phylogeny

**Figure 6:**
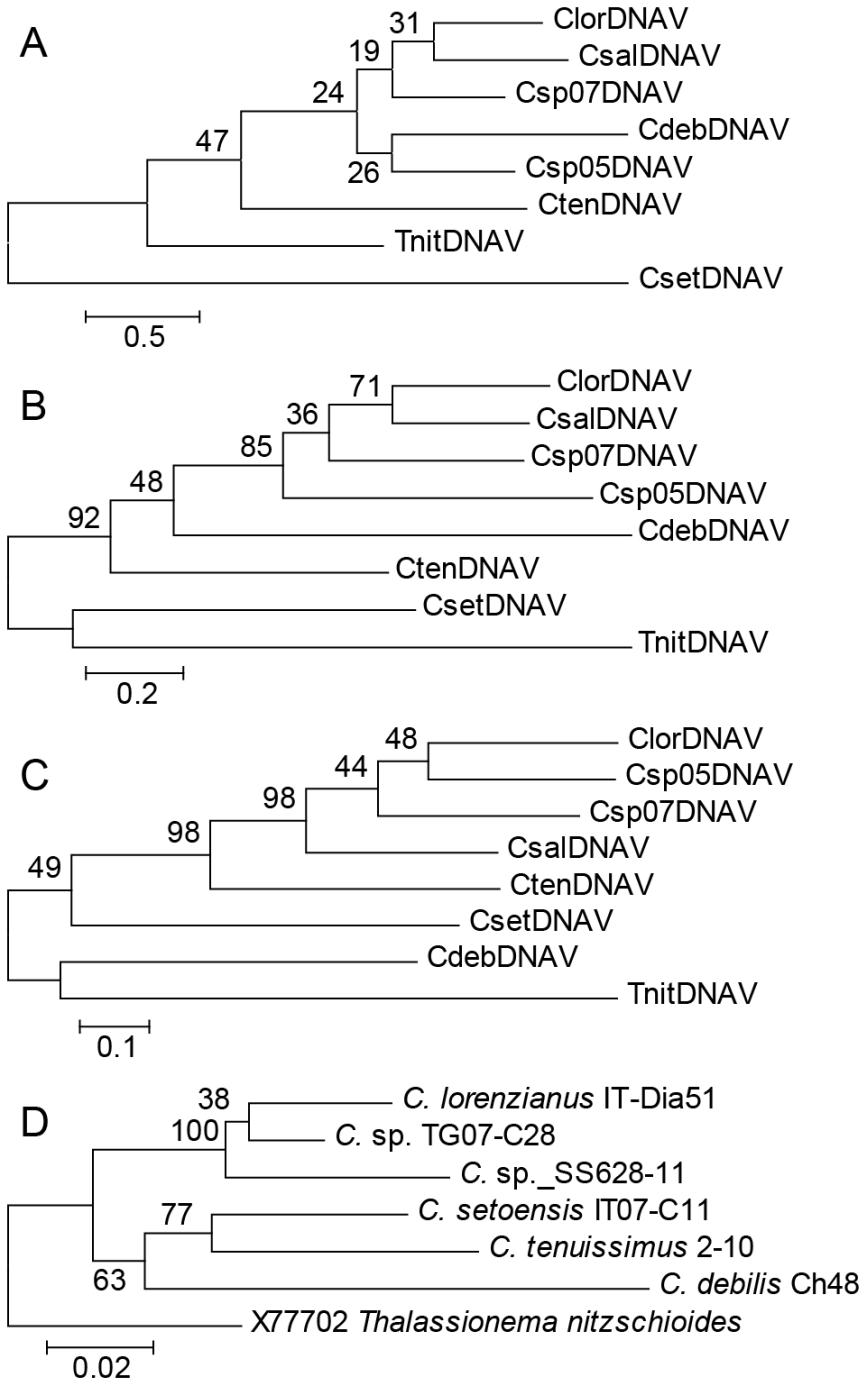
Phylogenetic analysis of *Bacilladnavirus* and host diatoms. (A–C) Maximum likelihood (ML) trees based on deduced amino acid sequences of ORFs. Bootstrap values (%) from 1,000 samples are shown at the nodes. The ML distance scale bars are shown. (A) ML tree by VP1. (B) ML tree by VP2. (C) ML tree by VP3. (D) Neighbor-joining tree for 18 s rRNA sequences of host diatoms against each diatom-infecting DNA virus.

ML methods were used to assess the phylogenetic relationship among the diatom ssDNA virus VP3 proteins that have higher similarities to those of other diatom viruses. The monophyly of Csp07DNAV, ClorDNAV, CsalDNAV, Csp05DNAV, and CtenDNAV was supported by a high bootstrap value (Fig. 6C). The results of the phylogenetic analyses also indicated that Csp07DNAV belongs to the genus *Bacilladnavirus*, whereas TnitDNAV, CdebDNAV, and CsetDNAV, which are other DNA viruses that infect diatoms, each might belong in separate groups. In contrast, the phylogenetic tree of proteins VP1 and VP2 showed the possibility that CdebDNAV belongs to the group containing Csp07DNAV, ClorDNAV, CsalDNAV, Csp05DNAV, and CtenDNAV. Especially, the monophyly of these viruses was supported by a high bootstrap value in the case of VP2. In the trees of each VP, CsetDNAV was excluded from the group of other centric DNA viruses that infect diatoms. The morphologic and genomic features of centric diatom DNA viruses are similar to each other except for CsetDNAV, which harbors multiple complementary fragments in its genome (Table 2). We should therefore reconsider the definition of the genus *Bacilladnavirus* based on information for diverse diatom viruses that harbor different genomic sequences and architectures in other locations.

Host diatom 18 s rRNA was detected as followed; *C. lorenzianus* strain IT-Dia51, AB847414; *Chaetoceros* sp. strain TG07-C28, AB847415; *C.* sp. strain SS628-11, AB847416; *C. tenuissimus* strain 2-10, AB847417; *C. setoensis* strain IT07-C11, AB847418; *C. debilis* strain Ch48, AB847419. The phylogenetic relationship for the host diatom was analyzed based on 18 s rRNA by using the ML method (Fig. 6D). The results showed that the phylogenetic relationships between the host diatoms and their infectious viruses are different each other. Evolutionary relationships between the diatom hosts and viruses might be an interesting topic for future studies.

Phylogenetic analysis showed that the tree for VP2, which encodes a putative viral structural protein, has a different conformation from that for VP3, which is a putative replication-related protein. The amino acid sequences of VP3 might be most suitable for conducting a phylogenetic test because they are highly similar among ssDNA diatom viruses. Since the viral capsid protein must have a significant relationship with virus host receptors, it is also an important component for understanding the evolution of this group. For further classifications of ssDNA diatom viruses, research should be focused on the capsid protein regions as well as replication proteins.

### Replication

**Figure 7:**
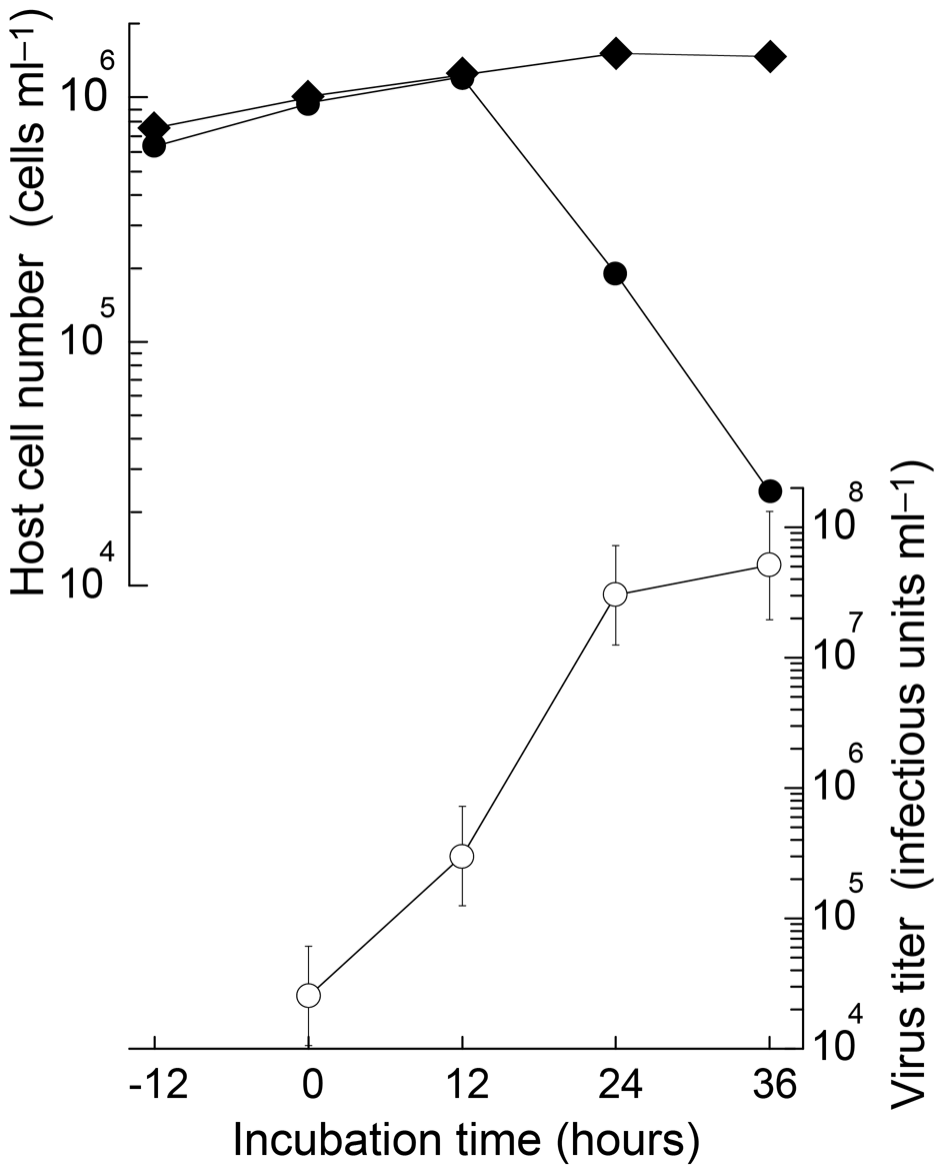
Growth experiments. Changes in the cell density of *Chaetoceros* sp. strain SS628-11 with inoculation (closed circles) or without virus inoculation (closed argyle squares) and in viral titer (open circles). Virus inoculation was performed at 0 h in an exponentially growing host culture at a multiplicity of infection of 0.027.

In the growth experiment, the *C.* sp. SS628-11 strain grew after 12 h post inoculation (hpi) in both the control- and virus-inoculated cultures. However, the cell number in the inoculated culture rapidly decreased after 24 hpi (Fig. 7). A significant increase was observed in the viral titer after 12 hpi (Fig. 7). Thus, the latent period of Csp07DNAV was estimated to be <12 h.

The average host/virus ratio, which ranged from 12 to 24 hpi, was used to calculate the burst size, which was estimated to be 29 infectious units•cell^−1^. The burst sizes of the previously reported ssDNA diatom viruses range between ∼10^1^ and ∼10^4^ infectious units•cell^−1^ [15], [16], [27]. The diversities of the burst sizes might reflect differences in the ecological strategy of each ssDNA virus species. In future studies, burst sizes of the viruses should be determined under various environmental conditions, *e.g.*, nutrients, salinity, and temperature, to assess the effects of the diatom viruses on host population dynamics.

Studies on diatoms and their infectious virus are still in their infancy. To understand the impact of viruses on diatoms and their ecological and evolutionary relationships in nature, further isolation and characterization of diatom viruses are necessary.
